# Cystic *papillary* adenoma of the seminal vesicle

**DOI:** 10.1186/s12894-021-00830-7

**Published:** 2021-04-15

**Authors:** B. Heijkoop, D. Bolton, D. Katz, Andrew Ryan, J. Epstein, S. Appu

**Affiliations:** 1 Austin Health, Melbourne, Australia; 2 Men’s Health Melbourne, Melbourne, Australia; 3 Tissupath, Melbourne, Australia; 4 Johns Hopkins Hospital, Baltimore, USA; 5 Department of Surgery, Monash University, Melbourne, Australia

**Keywords:** Cystic papillary adenoma, Seminal vesicle, Histology, Case report

## Abstract

**Background:**

Primary Seminal Vesicle (SV) tumours are a rare entity, with most SV masses representing invasion of the SV by malignancy originating in an adjacent organ, most often the prostate. Previously reported primary SV epithelial tumours have included adenocarcinoma and cystadenoma, with limited prior reports of inracystic papillary structures.

**Case presentation:**

A 35-year-old male presented with azoospermia, intermittent macroscopic haematuria, and mild right iliac fossa and groin pain. A papillary appearing seminal vesicle mass was found on imaging and seminal vesicoscopy. The mass was robotically excised with diagnosis of benign cystic papillary adenoma made.

**Conclusion:**

In this manuscript we describe a rare case of a benign cystic papillary adenoma of the seminal vesicle, a unique histological entity differentiated from cystadenoma of the Seminal Vesicle by its papillary component.

## Background

Primary Seminal Vesicle (SV) tumours are a rare entity, with most SV masses representing invasion of the SV by malignancy originating in an adjacent organ, most often the prostate. Primary SV tumours can be considered in groups according to their origins from epithelial, mesenchymal or other tissues, or from an infectious or inflammatory cause. Other rare SV entities that may need to be considered in the differential diagnosis include borderline Mullerian type serous neoplasms. Overall, benign aetiologies are less common than a malignant cause.

Previously reported primary SV epithelial tumours have largely included adenocarcinomas and cystadenomas, with few prior papers describing intracystic papillary structures [1–8]. We report a rare case of a benign cystic papillary adenoma of the seminal vesicle, representing a unique histological entity differentiated from cystadenoma of the SV by its papillary component.

## Case presentation

**Fig. 1:**
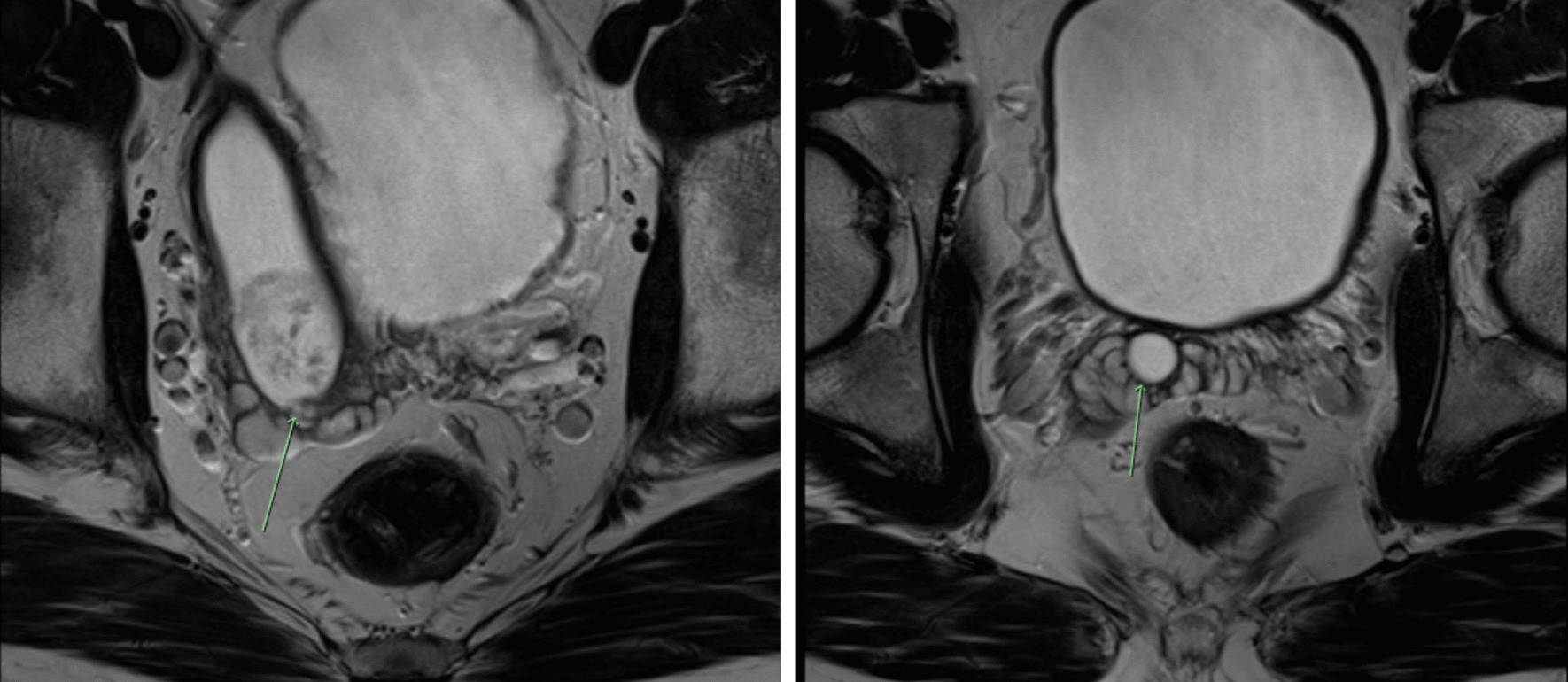
MRI images demonstrating cystic papillary adenoma of Seminal Vesicle

A 35 year old male was referred with an 8 month history of intermittent macroscopic haematuria, associated with mild right sided iliac fossa and groin pain. Past medical history included a prior right inguinal hernia repair and orchidopexy for undescended testis. The patient was also found to have azoospermia and was undergoing assessment in preparation for In vitro fertilization (IVF)/Intracytoplasmic Sperm Injection (ICSI). Examination of genitalia, testes and prostate was essentially unremarkable outside of an old right inguinal scar consistent with the history of orchidopexy and hernia repair. There was some thickening of the tissues around his right testicle consistent with previous orchidopexy. Initial workup of haematuria was performed. Urine microscopy demonstrated nonglomerular microscopic haematuria, cytology revealed atypical cells and the computerized tomography (CT) intravenous pyelogram (IVP) reported normal appearance of the upper urinary tract and bladder. However, it also described the incidental finding of a 7 × 3.5 × 2 cm fluid density mass at the posterior aspect of the bladder continuous with the right seminal vesicle. Blood tests were unremarkable including a PSA of 0.6 ng/ml. The seminal vesicle mass was further investigated with magnetic resonance imaging (MRI) which reported two cystic structures adjacent the bladder and appearing related to the seminal vesicle (Fig. 1). The larger structure terminated at the level of the verumontanum appearing to be a Müllerian duct remnant, and comment was made on the appearance of debris within the smaller structure. Operatively blood stained debris was seen at the verumontanum meatus and a rigid ureteroscope was used to perform seminal vesiculoscopy to inspect the lesion. A papillary mass was visible macroscopically and biopsies were taken of the mass. The biopsy histology noted atypical epithelial lesion with papillary features. Following discussion of these findings the patient elected to proceed and the cystic structure was excised via a robotic approach and sent for histopathology. The patient made a smooth recovery with normal ejaculation function and volume. The urine cytology also normalized post removal of the seminal vesicle.

**Fig. 2:**
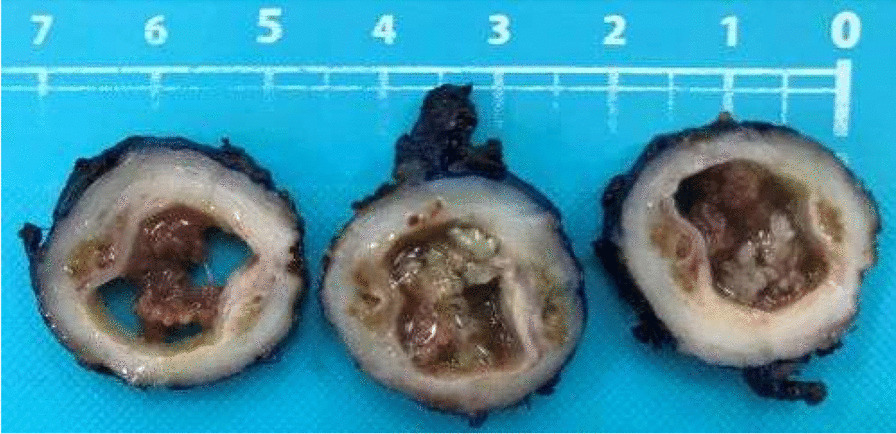
Serial transverse sections of right seminal vesicle – Cystic papillary adenoma of the seminal vesicle; with luminal dilation and intraluminal papillae

**Fig. 3:**
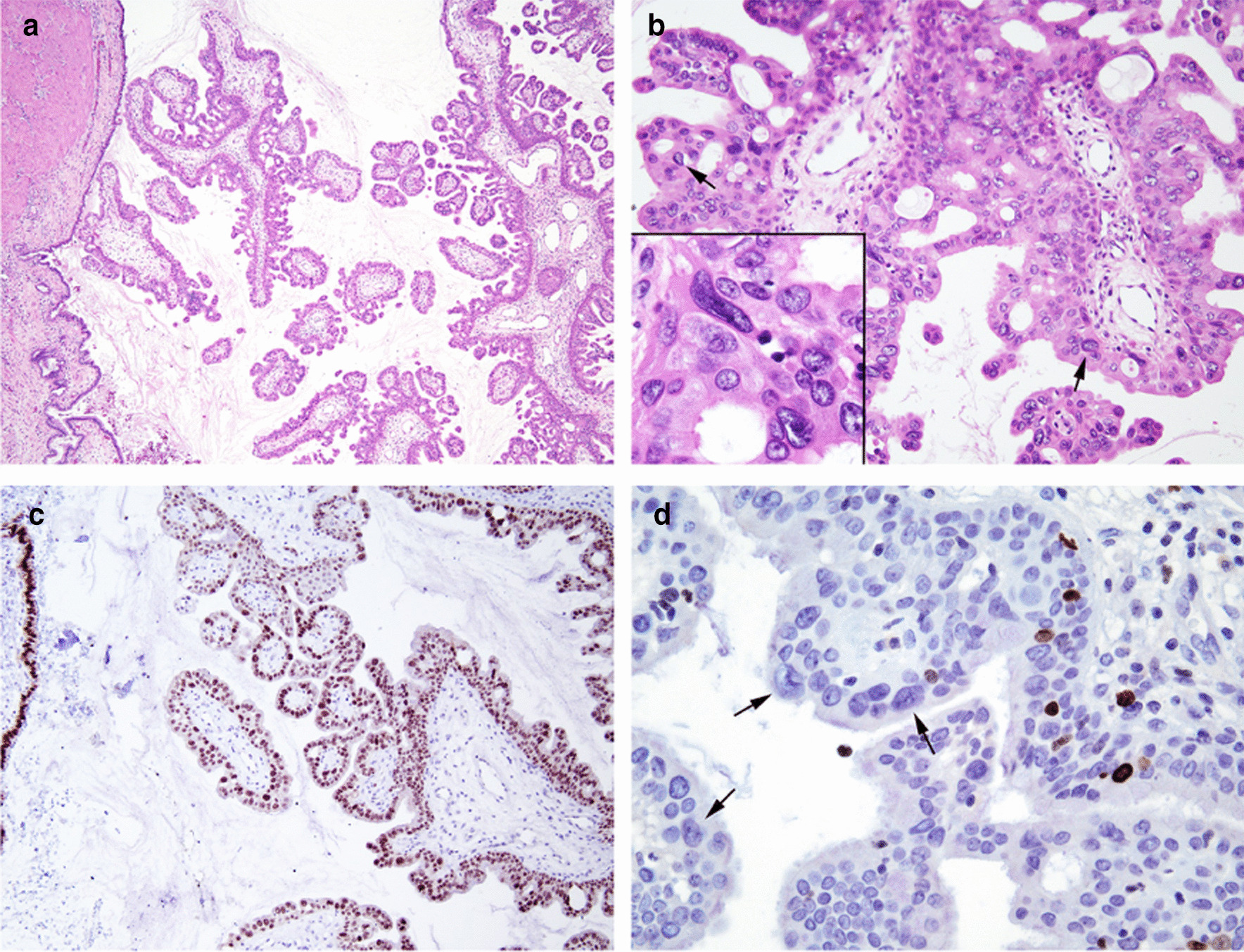
**a** Low magnification of cystadenoma arising in the seminal vesicle. The seminal vesicle wall and lining is at the left side of the image. **b** Higher magnification of cribriform and papillary growth pattern with most cells showing bland morphology with scattered larger atypical nuclei with a degenerative appearance (arrows). Inset shows degenerative atypia with smudgy chromatin lacking mitotic activity. **c** PAX 8 immunoreactivity in the normal seminal vesicle epithelium (left) and in the cystadenoma. **d** Ki67 showing scattered positive cells, but the larger cells with degenerative atypia (arrows) show a lack of ki67 staining

Macroscopically, the right seminal vesicle contained a friable tan-brown colored lesion measuring 15 × 13 mm (Fig. 2). On microscopy, the seminal vesicle lumen was dilated up to 15 mm containing several well developed organized papillary structures, some of which were infarcted. Both the cyst and papillae were lined by benign seminal vesicle and focal benign squamous and mucinous type epithelium. While cytological atypia was present in the seminal vesicle epithelium this was degenerative in nature with inconspicuous mitotic activity and a low Ki-67 index. Staining was positive for CK7, CK903, Ca-125 and PAX-8 and negative with PSA and PSAP, a typical staining pattern for seminal vesicle epithelial neoplasms (Fig. 3).

Given the atypical findings the specimen was referred to Uropathologist Professor Jonathan Epstein at the Johns Hopkins Hospital (Baltimore, USA) where a final diagnosis was made as a benign Cystic Papillary Adenoma of the seminal vesicle, which was considered a unique, distinct entity from cystadenoma of the seminal vesicle, which lacks the papillary component seen in this case.

The patient will continue with IVF workup and treatment. His MRI and urine cytology will be repeated 6-monthly for 2 years as a precaution prior to discharge.

## Discussion and conclusion

Given the unique histological findings we conducted a literature review, searching PubMed and EMBASE databases with the search terms Cystic Papillary Adenoma and Seminal Vesicle (all fields). Ultimately, despite the broad search strategy, only limited prior reports of intracystic papillary structures were identified [7, 8].

This raises several issues for the ongoing management of the patient. Firstly, is there a relationship between the seminal vesicle mass and the patient’s presenting problems of azoospermia, macroscopic haematuria and right sided abdominal and groin pain? While these initially appeared to be unrelated pathologies, it is not inconceivable that the SV mass may explain all three complaints; being of a significant size that could cause pain and a degree of obstruction to normal passage of sperm into the ejaculate, and vascular with potential to intermittently bleed. We anticipate further clinical review of symptoms, repeated urine microscopy and semen analyses would demonstrate resolution following excision of the SV mass if it is the cause. We do recommend the patient and partner continue with IVF treatment as planned in the interim to maximise the likelihood of achieving a healthy pregnancy in the event azoospermia persists, especially considering that the unilateral nature of the SV mass makes the hypothesis of an exclusively obstructive cause of azoospermia somewhat less likely.

Secondarily while the lesion is reported to be entirely benign and excised in its entirety with clear margins, with limited prior reports of this pathology there is no definite existing evidence on which to base further follow up. Given other benign papillary pathologies of the lower urinary tract such as papillary urothelial neoplasm of low malignant potential (PUNLMP) are known to have the potential to recur, and the potential relationship of this mass to the patient’s clinical presentation, we believe ongoing surveillance is warranted but optimal modality, frequency and duration are unclear. Initially we recommend repeating urine microscopy, cytology and imaging with MRI or CT on a six monthly basis. Surveillance could subsequently be performed annually if all investigations remain normal, with discharge to a general practitioner considered at 10 years post treatment without recurrence. However, should the patient develop recurrence of macroscopic haematuria or other symptomatology they should be thoroughly re-investigated with urine cytology, CT, IVP and cystoscopy, and we stress that the benign diagnosis of cystic papillary adenoma of the SV should not falsely reassure the clinician of the absence of more sinister diagnoses, potential recurrence or progression to a more aggressive lesion.

Finally, the use of a rigid ureteroscope to endoscopically visualize the mass was a useful component of initial investigation in this case. Consequently, while a technically challenging procedure, we propose that in the hands of an experienced endourologist, this technique may be useful in evaluation of similar presentations in future.

## Data Availability

All data generated and analysed during this study are included in the published article.

## Abbreviations

CT: Computed tomography
ICSI: Intracytoplasmic sperm injection
IVF: In vitro fertilization
IVP: Intravenous pyelogram
MRI: Magnetic resonance imaging
PSA: Prostate specific antigen
SV: Seminal vesicle
USA: United States of America
